# Does Having Rheumatoid Arthritis Increase the Dose of Depression Medications? A Mendelian Randomization Study

**DOI:** 10.3390/jcm12041405

**Published:** 2023-02-10

**Authors:** Xianjie Wan, Jiale Xie, Mingyi Yang, Hui Yu, Weikun Hou, Ke Xu, Jiachen Wang, Peng Xu

**Affiliations:** Department of Joint Surgery, Honghui Hospital, Xi’an Jiaotong University, Xi’an 710054, China

**Keywords:** rheumatoid arthritis, depression medications, causal association, genome-wide association study, Mendelian randomization

## Abstract

Background: Rheumatoid arthritis (RA) increases the risk of depression. However, studies on the effects of RA on the dose of depression medications are limited. Therefore, in this study, we used two-sample Mendelian randomization (MR) to explore whether RA increases the dose of depression medications and gain a more comprehensive understanding of the relationship between RA and depression. Methods: Two-sample MR was used to evaluate the causal effect of RA on the dose of depression medications. The aggregated data on RA originated from extensive genome-wide association studies (GWASs) of European descent (14,361 cases and 42,923 controls). The summary GWAS data for the dose of depression medications were derived from the FinnGen consortium (58,842 cases and 59,827 controls). Random effects inverse-variance weighted (IVW), MR-Egger regression, weighted median, and fixed effects IVW methods were used for the MR analysis. Random effects IVW was the primary method. The heterogeneity of the MR results was detected using the IVW Cochran’s Q test. The pleiotropy of the MR results was detected using MR-Egger regression and the MR pleiotropy residual sum and outlier (MR-PRESSO) test. Finally, a leave-one-out analysis was performed to determine whether the MR results were affected by a specific single-nucleotide polymorphism (SNP). Results: The primary method, random effects IVW, revealed that genetically predicted RA had a positive causal association with the dose of depression medications (Beta, 0.035; 95% confidence interval (CI), 0.007–0.064; *p* = 0.015). The IVW Cochran’s Q test results revealed no heterogeneity in the MR analysis (*p* > 0.05). The MR-Egger regression and MR-PRESSO tests revealed that there was no pleiotropy in our MR analysis. The leave-one-out analysis confirmed that a single SNP did not affect the MR results, indicating the study’s robustness. Conclusion: Using MR techniques, we discovered that having RA increases the dose of depression medications; however, the exact mechanisms and pathways still need to be further explored.

## 1. Introduction

Depression, often known as depressive disorder, is one of the most frequent emotion-related psychiatric illnesses in clinical practice, with typical symptoms including decreased interest, poor cognitive function, and affective disorders [1]. According to statistics, the lifetime prevalence of depression is 13.2%, and approximately 25% of women and 10% of men suffer from depression [2]. The pathogenesis of depression is complex, involving biological, physiological, and social circumstances, and can develop alone or in conjunction with other disorders. At this stage, it is believed to be closely related to inflammation and cytokines [3,4], monoamine neurotransmitters and their receptors [5], neurotrophic factors [6,7], and the hypothalamic–pituitary–adrenal (HPA) endocrine axis [8]. The treatment of depression mainly involves psychological counseling and medication, but the results are often unsatisfactory [9]. Depression can seriously affect patients and society. It is mainly manifested as a significant decline in all human functions, interfering with normal activities in the work, family, and social spheres; leading to severe pain and suffering; and even leading to a variety of serious problems, such as patients committing suicide [10]. Sufficient attention should be paid to physical and mental disorders associated with the patient’s clinical development.

Rheumatoid arthritis (RA) is a chronic inflammatory autoimmune disease [11]. RA mainly involves joints and is often accompanied by persistent joint pain, swelling, stiffness, and other symptoms [12]. However, RA not only damages the joints but also includes many extra-articular manifestations, such as rheumatoid nodules, pulmonary involvement or vasculitis, and systemic comorbidities, particularly vascular and metabolic effects [12,13,14]. The global average incidence of RA is estimated to be 0.5–1.0% [11]. Although RA can be relieved with strict lifestyle management and drug therapy, there is currently no cure [15]. As a kind of cumulative multi-system and multi-organ autoimmune disease, RA seriously endangers the health of patients and reduces the quality of life of patients [16]. It is precisely because of the above characteristics of RA that the symptoms caused by RA can affect the physical and mental health of patients, and even the diagnosis of RA may affect mental health [17]. Studies have found a link between RA and Alzheimer’s disease (AD) [18]. In addition, anxiety and depression are the most common mental manifestations of RA, and these psychiatric manifestations, such as anxiety and depression, have been strongly correlated with the severity of RA [19]. According to research statistics, the prevalence of depression in patients with RA is as high as 9.5–41.5% [20]. As an important means of treating depression, drug therapy can reflect the condition to a certain extent. Although the results of some observational studies reflect a partial relationship between having RA and the dose of depression medications to some extent, these observational studies are often disturbed by some confounding factors during the research process, and it is difficult to clearly conclude a causal relationship between the two. To clarify the causal relationship between having RA and the dose of depression medications, we still need a new research method to explore it.

The Mendelian randomization (MR) approach uses genetic variations as instrumental variables (IVs) to model and then makes causal inferences about exposures and outcomes at the genetic level. This approach can effectively avoid the problem of confounding factors influencing causal inference in traditional epidemiological studies, which can greatly improve the accuracy and credibility of conclusions. Currently, MR methods are widely used in studies examining the causal relationship between exposure factors and outcomes at the genetic level. In an MR study conducted to investigate the causal relationship between RA and cardiovascular disease, a significant genetic association was found between them [21]. In another MR study, RA was found to reduce the risk of Parkinson’s disease [22]. In examining the relationship between RA and AD, MR analysis supported a causal relationship between RA and a reduced risk of AD [23]. Some studies have also found that physical activity is associated with a reduced risk of depression [24]. In exploring the association of inflammation with depression and anxiety through MR, inflammation was found to be associated with the core depressive symptoms of low mood and lack of pleasure [25]. MR methods can better investigate the causality associated with their respective domains at the genetic level, and we attempted to use MR to investigate whether RA increases the dose of depression medications in order to provide a more comprehensive understanding of the relationship between RA and depression and to provide new ideas for future studies.

## 2. Materials and Methods

### 2.1. Study Design

**Figure 1:**
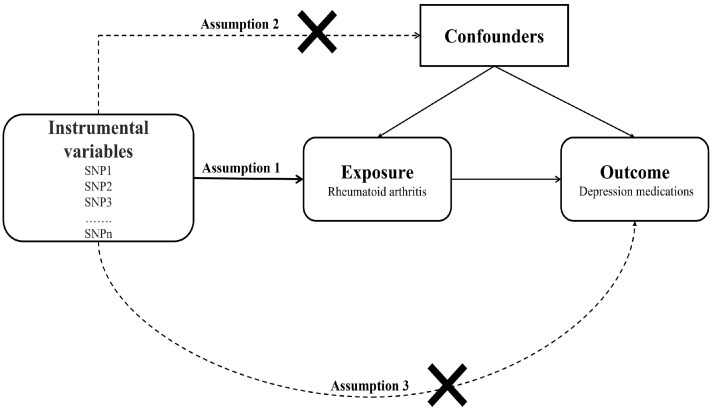
An overview of the study design. SNP, single-nucleotide polymorphism.

In the present study, SNPs were used as IVs to explore the causal relationship between RA (exposure) and the dose of depression medications (outcome). The experimental design is shown in Figure 1. The successful implementation of MR analysis requires that three assumptions be satisfied: (1) all the selected IVs must be strongly correlated with the exposure; (2) all selected IVs are independent of confounding factors; (3) none of the selected IVs influence the outcome through pathways beyond the exposure.

### 2.2. Data Resources

The data used in this study were obtained from the IEU Open GWAS database (https://gwas.mrcieu.ac.uk/ (accessed on 28 November 2022)), and more information on the data sources is presented in Table 1. For RA, summary-level data were derived from a large GWAS that included 57,284 individuals of European descent (14,361 cases and 42,923 controls) (GWAS ID: ebi-a-GCST002318). All patients with RA met the 1987 American College of Rheumatology criteria for RA diagnosis or were diagnosed by a specialist rheumatologist. More details can be found in a previously published paper [26]. For the dose of depression medications, summary-level GWAS data were obtained from the FinnGen consortium (https://www.finngen.fi/fi; accessed on 11 November 2022). FinnGen is a large public–private partnership that collects and analyzes genome and health data from over 300,000 Finnish participants. The dataset contained 58,842 cases and 59,827 controls of Finnish ancestry (FREEZE 5) (GWAS ID: finn-b-ANTIDEPRESSANTS). A total of 16,379,737 SNPs were identified. All cases were defined using the code M13 of the International Classification of Diseases, Tenth Revision.

### 2.3. Selection of IVs

We performed a series of quality control steps based on the GWAS summary results to select eligible IVs. First, SNPs that reached genome-wide significance (*p* < 5 × 10^−8^) were used as IVs for RA. Second, we removed linkage disequilibrium (LD) with r^2^ = 10,000 and kb = 10,000 to obtain independent SNPs. Third, we manually searched for secondary phenotypes that could potentially affect depression medications using the PhenoScanner database (http://www.phenoscaner.medschl.cam.ac.uk (accessed on 7 December 2022)). SNPs associated with secondary phenotypes were excluded, and the remaining SNPs were used for further MR analyses. Fourth, SNPs satisfying the above three steps were sought from the outcome dataset. We did not search for proxy SNPs that could not be identified in the outcome database. Fifth, we examined whether the selected SNPs were directly and significantly relevant to the outcome (*p* < 5 × 10^−8^ in the outcome datasets). Sixth, incompatible alleles and palindromic SNPs with intermediate allele frequencies were excluded from the MR analysis. Finally, the F statistics of these IVs were calculated to avoid bias triggered by weak IVs. F statistics of >10 indicate robust IVs [27]. The F statistics were calculated using the admittedly reliable formula F = R^2^ (N – K − 1)/(1 − R^2^)K [28,29]. R^2^ was calculated using the formula R^2^ = (2 × EAF × (1 − EAF) × Beta^2^)/[(2 × EAF × (1 − EAF) × Beta^2^) + (2 × EAF × (1 − EAF) × N × SE^2^)] [30], where R^2^, N, EAF, Beta, SE, and K refer to the cumulative explained variance of selected SNPs in the exposure, the sample size for the exposure GWAS, the effect allele frequency, the estimated effect on the exposure, the standard error of the estimated effect, and the number of IVs, respectively.

### 2.4. Statistical Analyses

Multiple methods were used to assess the causal effect of RA on the dose of depression medications (including random effects IVW, weighted median, MR-Egger regression, and fixed effects IVW methods). The random effects IVW method was the primary method. The random effects IVW method uses a meta-analytical approach to combine the Wald ratios for the causal effects of each SNP and can provide the most precise estimates when all selected IVs are valid [31,32]. The weighted median method can provide a suitable causal assessment even if more than 50% of the IVs are ineffective [33]. The MR-Egger regression method provides an estimate corrected for multiple effects, but its main shortcoming is its lack of power [34]. Compared with the MR-Egger regression, the weighted median method improves accuracy and is more robust to irregularities in causal effects [35]. The fixed effects IVW method provides a better causal assessment than the random effects IVW method when the results are not heterogeneous [36].

### 2.5. Sensitivity Analysis

Various sensitivity analysis methods were used in this study. The IVW estimates approach was used to detect heterogeneity, which was measured using Cochran’s Q statistic and a *p*-value > 0.05, suggesting no heterogeneity [37]. The MR-Egger regression method was used to test for directional pleiotropy based on the intercept test [38]. Because MR-Egger may show low accuracy under certain conditions, the MR pleiotropy residual sum and outlier (MR-PRESSO) method was also used to test outliers and pleiotropy, and a Global Test *p*-value of >0.05 suggested no potential pleiotropy [39]. Outliers in an IVW analysis can be detected and corrected using the MR-PRESSO test [40]. Finally, a leave-one-out analysis was conducted to examine whether a single SNP influenced the causal relationship between RA and the dose of depression medications.

All statistical and sensitivity analyses were performed using the “TwoSampleMR” (version 0.5.6) and “MR-PRESSO” packages in R software (version 4.2.1).

## 3. Results

### 3.1. Selection of IVs

After screening SNPs with a condition of *p* < 5 × 10^–8^ and removing the LD, 60 SNPs were identified as IVs for RA. Next, we manually searched for the second phenotype using the PhenoScanner database. Two SNPs (rs2561477 and rs2736337) related to worry or anxiety were removed because they were potential confounders for the dose of depression medications [41,42]. When SNPs could not be extracted from the outcome GWAS dataset, we did not search for proxy SNPs. Five palindromic SNPs (rs13330176, rs168962, rs74956615, rs909685, and rs9296009) with moderate allele frequencies were deleted. Finally, the 47 remaining SNPs were used for the MR analysis. The F statistic of the selected SNPs ranged from 27 to 459, suggesting that they were robust IVs (supplementary Table S1).

### 3.2. MR Analysis

**Figure 2:**
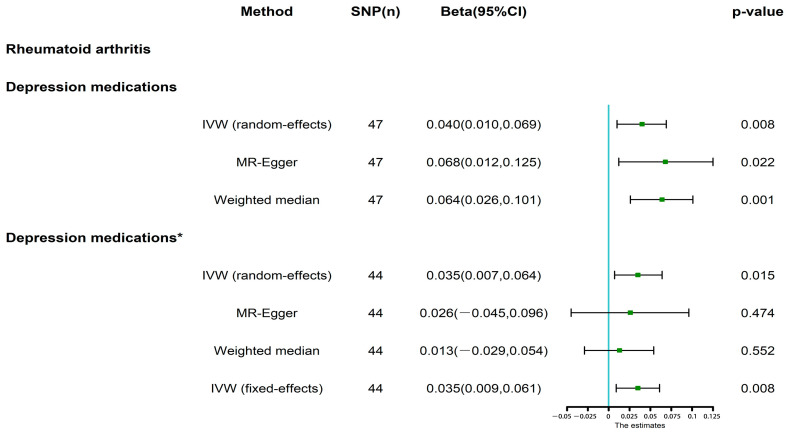
The results of Mendelian randomization for the associations of genetically predicted rheumatoid arthritis and rheumatoid arthritis * (after removing outliers) with depression medications.

**Figure 3:**
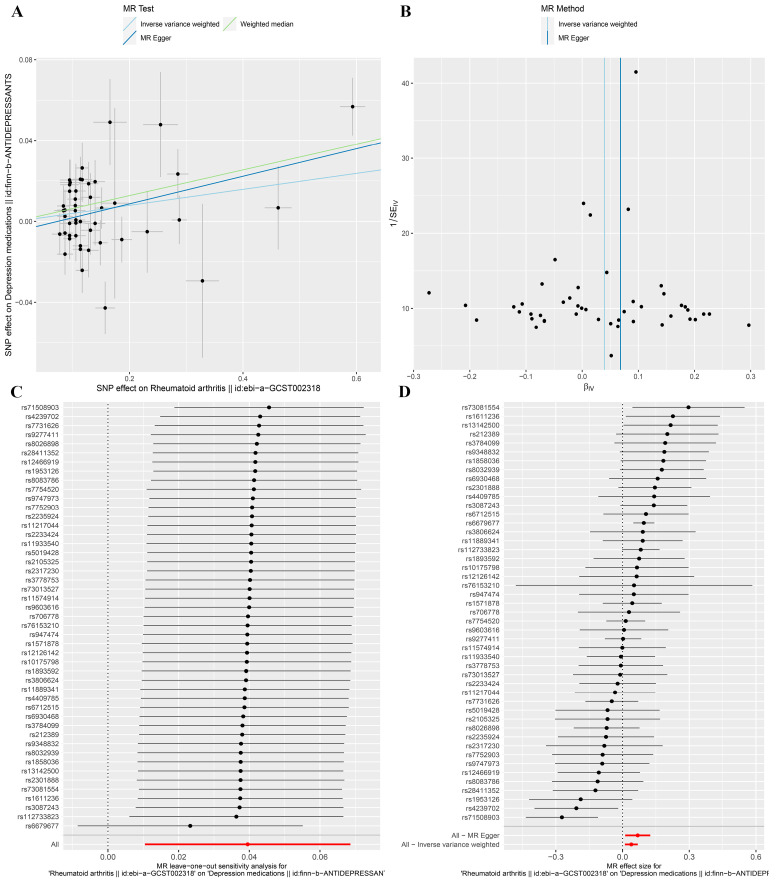
Association between genetically predicted rheumatoid arthritis and depression medications presented in (**A**) scatter plot, (**B**) funnel plot, (**C**) leave-one-out sensitivity analysis, and (**D**) forest plot.

The random effects IVW method showed that genetically predicted RA (Beta, 0.040; 95% CI, 0.010–0.069; *p* = 0.008) (Figure 2 and Figure 3A,D) was positively associated with the dose of depression medications. The MR-Egger and weighted median methods both showed results consistent with the random effects IVW (Figure 2). A sensitivity analysis was conducted to verify the reliability of the results. The IVW Cochran’s Q test revealed that our results were affected by heterogeneity (Cochran’s Q = 77.72, *p* = 0.002) (Figure 3B, Table 2). Furthermore, the MR-Egger test (intercept = −0.005, *p* = 0.251) showed no multiple effects in our MR analysis (Figure 3A, Table 2). The MR-PRESSO test showed that the MR analysis results for RA and the dose of depression medications had potential pleiotropy and outliers (rs4239702, rs6679677, and rs71508903) (Global Test *p*-value = 0.002) (Table 2). The leave-one-out analysis indicated that our MR analysis results were not affected by any single SNP (Figure 3C).

### 3.3. MR Analysis after Removing Outliers

**Figure 4:**
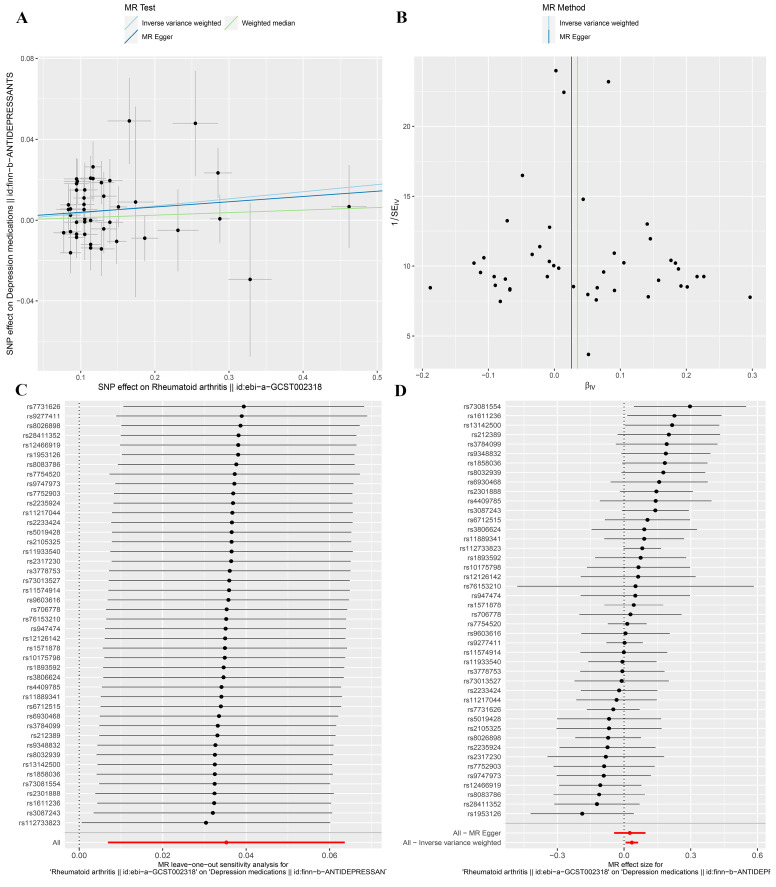
Association between genetically predicted rheumatoid arthritis (after removing outliers) and depression medications presented in (**A**) scatter plot, (**B**) funnel plot, (**C**) leave-one-out sensitivity analysis, and (**D**) forest plot.

Owing to the existence of outliers and potential pleiotropy, we removed the outliers to perform the MR analysis again. After removing outliers, the random effects IVW method revealed that genetically predicted RA was positively associated with the dose of depression medications (Beta, 0.035; 95% CI, 0.007–0.064; *p* = 0.015) (Figure 2 and Figure 4A,D), whereas the MR-Egger (Beta, 0.026; 95% CI, −0.045–0.096; *p* = 0.474) and weighted media (Beta, 0.013; 95% CI, −0.029–0.054; *p* = 0.552) methods revealed that genetically predicted RA was not associated with the dose of depression medications (Figure 2 and Figure 4A,D). Cochran’s Q test (Cochran’s Q = 51.35, *p* = 0.179) indicated the absence of heterogeneity in our MR analysis (Figure 4B, Table 2). Thus, the fixed effects IVW method provided a more accurate assessment of causality than the random effects IVW method did. As expected, the fixed effects IVW (Beta, 0.035; 95% confidence interval (CI), 0.009–0.061; *p* = 0.008) method also showed a positive association between RA and the dose of depression medications. (Figure 2). The MR-Egger (intercept = 0.001, *p* = 0.779) and MR-PRESSO (Global Test *p*-value = 0.179) methods confirmed that no horizontal pleiotropy (Figure 4A, Table 2) or outliers (Table 2) were included in our analysis. Finally, the leave-one-out analysis showed that the causal effect of RA on the dose of depression medications was not disproportionately influenced by a single SNP (Figure 4C), illustrating the reliability of our results.

## 4. Discussion

The present study excluded confounding factors and reverse causality by using MR methods, which gave higher accuracy and confidence to our results. We found that there is a causal relationship between having RA and the dose of depression medications at the genetic level and that having RA can lead to an increase in the dose of depression medications.

A series of previous observational studies have found a significant increase in the incidence of depression in patients with RA compared with that in the general population [43]. A study on the bidirectional association between RA and depression found a higher incidence of depression in patients with RA than in patients without RA [44]. In an epidemiological survey conducted in Iran, a very high prevalence of depression and anxiety was found in patients with RA [45]. Furthermore, patients with RA are more likely to have depressive symptoms when they are functionally constrained [46]. Poor quality of life and low educational attainment in patients with RA have been found to be significantly associated with anxiety/depression [47]. On the other hand, studies have reported that anxiety and depression are prevalent mental symptoms of RA and are closely associated with the severity of the disease [19]. Therefore, patients with RA may have a higher incidence of depression and a higher severity of depression, which leads to higher doses of depression medicines. However, an MR study showed different results than the findings of these observational studies [48]. Fang et al. found that RA did not lead to an increased risk of depression at the genetic level (OR, 0.999; 95% CI, 0.984–1.014; *p* = 0.932). The MR method used in this study treated genetic variations as IVs; first, the inheritance of each trait was independent of the inheritance of other traits and showed randomness, so the genotype of the offspring was unlikely to be related to environmental confounding factors in the population. Second, genotype distribution preceded the acquired exposure in time, and the link between the genotype and the disease was not affected by reverse causation. Third, exposure-related genotypes are often associated from birth to adulthood, so attenuation due to error can be avoided in causal inference. Limitedly, this MR study only confirmed that RA and depression have no causal relationship at the genetic level. It is significant to note that a number of factors, including inflammation, may mediate the high incidence of depression in RA patients and contribute to its development. Several studies showed that systemic inflammation in RA contributes to the occurrence of depression [49,50]. Accumulated evidence suggested that inflammation was critical to the link between RA and depression [51]. Thus, RA increases the risk of depression, and the severity of depression may depend on the severity of RA; the treatment of depression associated with RA may require taking higher doses of depression medications. Considering that increasing the dose of depression medications in patients with RA may be associated with the high incidence of depression in patients with RA and the severity of RA, the pathogenesis of depression in RA patients is very important.

The etiology of depression in RA patients is complex and varied, and it is generally believed that the diagnosis of RA, symptoms, loss of social roles, disability due to the disease, and medication side effects can all have an impact on it [51]. First, studies have revealed a substantial relationship between episodes of depression and the severity and duration of chronic pain, which is a symptom that is common in patients with RA [52]. Similarly, studies have found that depression in patients with RA is associated with more pain, fatigue, and impaired quality of life and that increased disease activity increases the risk of depression in RA [17]. Some studies have shown that pain, especially pain in multiple areas, is a risk indicator for depression and anxiety disorders [53]. Second, a study conducted in China found a significant relationship between education, quality of life, and anxiety or depression among Chinese patients with RA [47]. One study found that declining mental health was common in RA [54]. A study conducted in Singapore also found that poor mental health was associated with depression in RA [55]. Physical disability, helplessness, and passive coping can significantly affect the level of pain and depression experienced by people with RA [56]. Finally, RA is a disease characterized by an inflammatory response, and previous studies have found that inflammation may be the most important cause of depression in patients with RA. It has been suggested that pro-inflammatory cytokines (e.g., tumor necrosis factor, interleukin (IL)-1, IL-6, and IL-18) participate in the pathogenesis of RA and the development of depression in patients with RA [29]. Similarly, elevated serum IL-6 and IL-17 levels in patients with RA were shown to induce arthritis and cause emotional symptoms, especially depressive symptoms [30]. The specific pathways may be achieved in three ways [51]: (1) through the neural pathway of afferent nerves, thus affecting the hypothalamus; (2) through direct contact with the choroid plexus (no blood–brain barrier) and the organs around the ventricle, the impact on the nervous system is achieved through the humoral pathway; and (3) through activation of endothelial cells in the brain, promoting the activation of microglia and the secretion of pro-inflammatory cytokines, proteases, and chemokines, which can recruit monocytes to brain regions related to behavior [49]. When pro-inflammatory cytokines come into contact with the central brain, they can affect areas that are functionally altered in depression (e.g., the mid-frontal cortex, hippocampus, and basal ganglia), as well as neuroendocrine function (HPA axis) and neurotransmitter metabolism. However, there are also studies that have pointed out that levels of pro-inflammatory cytokines (IL-1β, IL-6, and TNF-α) in patients with RA are not associated with depression [57]. The etiology of depression in patients with RA is complex and unclear from previous studies, and more in-depth studies are needed.

Previous studies have found that RA increases the risk of depression through multiple pathways (symptoms, psychological factors, inflammation, etc.), and the severity of depression may depend on the severity of RA. Therefore, treating depression associated with RA may require taking higher doses of depression medications. However, this may only be part of the pathway, and the relationship between the two is complex and may be linked in more ways. Adding a genetic dimension not previously discussed, our study found a causal relationship between RA and the dose of depression medications at the genetic level (Beta, 0.040; 95% CI, 0.010–0.069; *p* = 0.008), and having RA can lead to an increase in the dose of medications for depression. According to the results of our study and previous studies, the dose variation of depression medications taken by RA patients with depression is not only related to symptoms, inflammation, etc., but there is a direct causal relationship between RA and the dose of depression medications at the genetic level.

Our study was the first to investigate whether RA increases the dose of depression medications through large-scale data analysis and using MR at the genetic level. However, there are some limitations to our study. First, our data were obtained from European databases, and the study population was exclusively European, leading to the need for caution when applying our findings to other populations. Second, RA is influenced in part by sex, but our study did not distinguish between men and women, and we need to take this into account when analyzing the dose of depression medication use in single-sex populations alone. Third, depressive medications are often used to treat neuropathic or chronic pain present in RA [58,59], which may have interfered with our results to some extent. Finally, our study only investigated whether RA significantly increased the dose of depression medications, and we should investigate the relationship between RA and depression to gain a more comprehensive understanding of this relationship.

## 5. Conclusions

In conclusion, we found that there is a causal relationship between having RA and the dose of depression medications at the genetic level and that having RA can lead to an increase in the dose of depression medications. This provides new evidence for future studies in this field at the genetic level and provides new ideas for future studies.

## Figures and Tables

**Table 1:** Detailed information on data sources in Mendelian randomization analysis.

Traits	GWAS ID	Sample Size	Number of SNPs	PMID	Author
Rheumatoid arthritis	ebi-a-GCST002318	57,284	9,700,598	24390342	Okada et al., 2014 [26]
Depression medications	finn-b-ANTIDEPRESSANTS	118,669	16,379,737		FinnGen consortium

GWAS, genome-wide association study; SNP, single-nucleotide polymorphism.

**Table 2:** The results of sensitivity analyses using multiple methods.

	MR-PRESSO	IVW Estimates		MR-Egger Pleiotropy Test	
Rheumatoid Arthritis	Global *p*-Value	Cochran’s Q	*p*-Value	MR-Egger Intercept	*p*-Value
Depression medications	0.002	77.72	0.002	−0.005	0.251
Depression medications *	0.179	51.35	0.179	0.001	0.779

MR-PRESSO, Mendelian randomization-pleiotropy residual sum and outliers; IVW, inverse-variance weighted; *, result of recalculation after removing outliers.

## Data Availability

The datasets supporting this study are available from IEU OpenGWAS (GWAS ID: ebi-a-GCST002318, https://gwas.mrcieu.ac.uk/ (accessed on 28 November 2022)) and the FinnGen consortium (https://www.finngen.fi/ (accessed on 11 November 2022), finn-b-ANTIDEPRESSANTS).

## Supplementary Materials

The following supporting information can be downloaded at: https://www.mdpi.com/article/10.3390/jcm12041405/s1, Table S1: Detailed statistics about selected IVs for causal effect of rheumatoid arthritis on depression medications.
