# Supplementary material for: Does Having Rheumatoid Arthritis Increase the Dose of Depression Medications? A Mendelian Randomization Study
Source: J Clin Med. 2023 Feb 10;12(4):1405. doi: 10.3390/jcm12041405 (PMC9961843; doi:10.3390/jcm12041405)
Supplement: Supplementary file 1 [file jcm-12-01405-s001.zip › jcm-2131634-supplementary.pdf]

SNP	effect_allele	other_allele	beta	se	R^2	F	sample size	pval
rs10175798	A	G	0.086	0.014	0.0007	39	57284	5.40E-09
rs11217044	C	T	-0.131	0.018	0.0009	52	57284	3.60E-15
rs112733823	T	C	0.285	0.019	0.0040	229	57284	6.60E-39
rs11574914	A	G	0.113	0.018	0.0007	40	57284	2.10E-13
rs11889341	T	C	0.131	0.018	0.0010	55	57284	6.70E-19
rs11933540	C	T	0.139	0.018	0.0011	61	57284	8.80E-17
rs12126142	A	G	-0.083	0.016	0.0005	26	57284	3.50E-09
rs12466919	T	C	0.113	0.013	0.0012	71	57284	5.70E-13
rs13142500	C	T	0.094	0.017	0.0005	30	57284	5.00E-09
rs1571878	T	C	-0.151	0.012	0.0029	165	57284	6.10E-30
rs1611236	A	G	-0.117	0.011	0.0018	106	57284	2.10E-15
rs1858036	G	A	-0.113	0.014	0.0012	67	57284	1.20E-14
rs1893592	C	A	-0.104	0.014	0.0010	56	57284	3.70E-12
rs1953126	C	T	-0.086	0.014	0.0006	37	57284	1.00E-09
rs2105325	C	A	0.105	0.017	0.0006	37	57284	3.10E-10
rs212389	A	G	0.095	0.018	0.0005	27	57284	3.30E-10
rs2233424	T	C	0.231	0.028	0.0012	70	57284	7.60E-19
rs2235924	A	G	-0.094	0.017	0.0006	32	57284	3.60E-09
rs2301888	A	G	-0.128	0.017	0.0010	56	57284	2.20E-18
rs2317230	T	G	0.077	0.014	0.0005	30	57284	2.10E-08
rs28411352	T	C	0.113	0.013	0.0012	71	57284	3.60E-12
rs3087243	A	G	-0.139	0.017	0.0011	65	57284	1.70E-22
rs3778753	G	A	0.105	0.017	0.0006	37	57284	1.10E-14
rs3784099	A	G	-0.094	0.017	0.0006	32	57284	7.10E-10
rs3806624	G	A	0.083	0.011	0.0010	55	57284	1.90E-08
rs4409785	C	T	0.105	0.017	0.0006	37	57284	3.00E-08
rs5019428	A	G	0.086	0.014	0.0007	39	57284	7.20E-10
rs6712515	C	T	-0.104	0.014	0.0010	56	57284	6.70E-15
rs6930468	G	A	0.094	0.011	0.0012	69	57284	5.50E-11
rs706778	T	C	0.086	0.014	0.0007	39	57284	1.50E-10
rs73013527	T	C	-0.094	0.017	0.0006	32	57284	9.80E-11
rs73081554	T	C	0.166	0.029	0.0006	32	57284	4.60E-08
rs76153210	T	C	0.174	0.025	0.0008	48	57284	2.20E-09
rs7731626	A	G	-0.186	0.018	0.0018	106	57284	7.30E-24
rs7752903	G	T	0.329	0.029	0.0022	127	57284	2.70E-26
rs7754520	T	C	-0.462	0.024	0.0066	379	57284	9.00E-67
rs8026898	A	G	0.148	0.013	0.0023	130	57284	6.50E-19
rs8032939	C	T	0.117	0.017	0.0008	44	57284	4.80E-16
rs8083786	G	A	0.128	0.018	0.0009	52	57284	1.00E-15
rs9277411	T	C	-0.288	0.013	0.0079	459	57284	1.50E-85
rs9348832	A	G	0.255	0.031	0.0012	69	57284	5.20E-19
rs947474	A	G	0.104	0.018	0.0006	33	57284	1.50E-08
rs9603616	T	C	-0.105	0.017	0.0007	40	57284	4.60E-12
rs9747973	T	C	-0.094	0.011	0.0013	72	57284	1.90E-12

Supplementary Table S1 Detailed statistics about selected IVs for causal effect of rheumatoid arthritis on depression medications.
